# “It is very hard to just accept this” – a qualitative study of palliative care teams’ ethical reasoning when patients do not want information

**DOI:** 10.1186/s12904-024-01412-8

**Published:** 2024-04-05

**Authors:** Joar Björk

**Affiliations:** 1 Centre for Research Ethics and Bioethics (CRB), Department of Public Health and Caring Sciences, Uppsala University, Uppsala, Sweden; 2 Department of Research and Development, Region Kronoberg, Växjö, Sweden; 3 Stockholm Centre for Healthcare Ethics (CHE), LIME, Karolinska Institutet, Stockholm, Sweden

**Keywords:** Palliative care, Autonomy, Health care staff, Qualitative research, Informed consent, Beneficence

## Abstract

**Background:**

The aim of this study was to explore how palliative care staff reason about the autonomy challenge that arises when a patient who has first said he wants full information appears to change his mind and rejects being informed.

**Methods:**

The study had a qualitative and exploratory design. Participants (physicians, registred nurses, social workers, physiotherapists and occupational therapists) were recruited from palliative care teams in southern Sweden. Six separate focus group interviews with a total number of 33 participants were conducted. The teams were asked to discuss a fictional case of a man who first wants, then rejects, information about his situation. The interviews were audiotaped and transcribed verbatim. Reflexive thematic analysis following Braun and Clarke was undertaken to analyse data.

**Results:**

The analysis resulted in three themes: *Patients have a right to reject information*, *Questioning whether this patient WANTS to reject information* and *There are other values at stake, too*. Although participants endorsed a right to reject information, they were unsure whether this right was relevant in this situation, and furthermore felt that it should be balanced against counteracting factors. The effect of such balancing was that participants would aim to find a way to present relevant information to the patient, but in a probing and flexible way.

**Conclusions:**

In their work with dying patients, palliative care staff meet many autonomy challenges. When faced with a choice to withhold information as per a patient’s wishes, or to provide information with the patient’s best interest in mind, staff find it hard to balance competing values. Staff also find it hard to balance their own interests against a purely professional stance. The overall strategy seems to be to look for caring ways to impart the information.

**Supplementary Information:**

The online version contains supplementary material available at 10.1186/s12904-024-01412-8.

## Background

Respect for patient autonomy is one of the central ethical principles or ideals in modern health care [1]. What it means to respect patients’ autonomy is a complex question, and the concept and implications of autonomy are much contended in contemporary bioethics [2–5]. Nonetheless, most agree that patients should be invited to participate as far as possible in medical decision-making and that medical information should be tailored according to the patient’s previous knowledge and expressed information preferences. The same consideration is manifest in many national healthcare jurisdictions. For instance, the Swedish Patient law stipulates that the patient should be given information about his/her medical situation and the expected future health trajectory, but also that if patients do not wish such information this should be respected [6].

Palliative care (PC) is the subdivision of care which is focused on patients with life threatening illness [7]. The World Health Organization emphasises that PC ”should be provided through person-centred and integrated health services that pay special attention to the specific needs and preferences of individuals” [ibid]. Many publications in and on PC stress the profound importance of autonomy as a guiding ethical ideal in this context [8, 9], although some also point out that ”standard” accounts of autonomy may not be suitably nuanced for PC [10]. Reasons for suggesting a special understanding of autonomy within PC include that PC patients are particularly ill, that families’ preferences play an especially important role in PC, and that some understandings of autonomy posit a relation of distrust between patient and healthcare provider [11–13].

Despite the great importance of patient autonomy as an ethical ideal in PC and elsewhere, it may sometimes be difficult to tease out which action would in fact best respect autonomy in a particular clinical situation [14, 15]. Practical and theoretical challenges regarding how to best respect autonomy may arise, for instance, in cases of patient ambivalence and intra-personal conflicts of interest [16–18], autonomy-promoting withholding of information [19] and advance directives [20]. Add to this that autonomy is not the only aspect to account for in the ethical calculus. Indeed, healthcare staff must seek to balance autonomy considerations with (at least) considerations about potential beneficence and harm, as well as potential direct and indirect effects on other patients [21].

There is to date very little understanding of how PC staff reason about and deal with tricky autonomy cases. A limited number of qualitative studies have been undertaken which shine a light on some aspects [see for instance 22–25], but much remains unclear. As part of a larger project on patient autonomy in PC^1^, the present study aimed to expand this knowledge by investigating how PC teams reflect upon a clinical situation where a patient first claims to want information about his likely health trajectory, but then reports he has changed his mind so that he no longer wishes to be informed. To gain an in-depth understanding of the ethical as well as practical aspects of such a situation, the study aimed to elucidate both how PC teams reason about the ethics, and how they claim they would usually act in such a situation.

## Methods

### Design

The study had a qualitative, exploratory and descriptive intent. Data was collected by focus group interviews, as this is a suitable way of getting information about factors which may influence motivation, opinions and behaviour in complex social settings [26]. Reflexive thematic analysis according to Braun and Clarke was chosen as method for data analysis as it may be used to understand how persons make sense of the world, which is relevant for a study involving ethical attitudes [27–29]. The method does not obscure but rather emphasises the creative input of the researcher, whereby both coding and the construction of themes is seen as a partly subjective process. In terms of Byrne’s four theoretical assumptions [27], this project was informed by a constructionist epistemology, meaning that language is seen not merely as an expression of experienced reality but also plays a role in constructing it. The orientation to data interpretation was mainly experiential as the study aimed to examine how participants understand certain professional challenges. Data analysis was inductive, data-driven and utilised open coding. In coding and analysis both semantic and latent content was interrogated.

### Participants

Participants were drawn from six specialized PC teams in three southern Swedish regions (Region Jönköping, Region Halland and Region Blekinge). By international standards these regions are sparsely populated, with some mid-sized urban areas, encompassing a total population of approximately 850 000 inhabitants. The PC is organized similarly in the three regions, with mobile teams attending to patients in their homes and in qualified nursing homes, as well as in hospitals. Driving times are long, up to 90 min one-way. The teams are multiprofessional, consisting of 10–20 members (typically physicians, nurses and social workers, although some teams also have physiotherapists and occupational therapists). Staff may or may not have formalized PC specialist qualification. For a fuller description about the PC services in this geographical area, see [30] where the teams studied here correspond to the “less developed PC” category in that article. The “less developed PC” setting was chosen for this study as the aim was to understand an everyday ethical approach to the challenge.

Recruitment started with an informative letter to the head of each PC team. The head of each team was asked to put together a group for interview with suitable variation regarding professional background, working experience, gender and age. Prior to commencing each interview the composition of the groups was checked to see that there was sufficient variation.

### Data collection

The vignette and the interview agenda (see below) were developed for this study. They were pilot tested in the author’s own work-place in Region Kronoberg (results not included in the study, but congruent with the results).

The focus group interviews were conducted in undisturbed rooms at the participants’ work places. JB was the sole interviewer. Each focus group interview commenced with JB reading a vignette to introduce the kind of challenge to be discussed:

*Mr B, a man with disseminated prostate cancer, is cared for by the specialized palliative care team. He has repeatedly stated to the team that it is vital to him to have full knowledge about his situation, including what his death may come to look like. As there have been many bodily symptoms to deal with, his questions about death and dying have not yet been answered. Now, the team has set aside time for the conversation Mr B has asked for. But as soon as they start talking, Mr B states that he does not want any more information and that this issue should not be brought up again. He provides no reason. Nothing in the situation appears to have changed. He is perceived to be of sound mind, now as before.*

A semi-structured interview agenda was used to make sure key topics were covered (do you recognise situations like this one; how do you as individuals and teams usually deal with situations like this; what are your feelings and reflections about the situation). (See also Supplementary file). During the interview, open questions were used to encourage participants to develop their answers, and participants were urged to discuss among themselves [26]. Field notes were taken immediately after each interview to aid in the analytical process. Interviews were audio recorded and transcribed verbatim.

Notions of saturation are contested within this field of qualitative research [31], where a pragmatic view of sample sizing is advocated. Instead a provisional sample size of six focus group interviews was estimated based on previous recommendations [see for instance 29, 32], and after having conducted six interviews the study was discontinued as it was felt the gathered data enabled the researcher to give a rich presentation of the studied phenomenon.

### Data analysis

In accordance with suggestions by Braun and Clarke, data analysis consisted of six phases [27, 28]. In *Phase 1 (Familiarisation)*, the entirety of the transcribed data corpus was read and reread with a curious mind and pen in hand to get a thorough understanding of the whole material. In *Phase 2 (Generation of codes)*, short labels summarizing relevant meaning units were created and refined. Data obviously not relevant to the research questions was left un-coded, whereas attention to both semantic and latent meaning meant that some data segments were double-coded. In *Phase 3 (Construction of candidate themes)* codes were grouped by identification of central organising concepts. Here, preliminary thematic maps were drawn to organise candidate themes as well as identify relations between them. In *Phase 4 (Review of candidate themes)* the suggested way of organizing information was reviewed and revised. Themes should be comprehensive as well as non-overlapping, and capture relevant meanings within the data. In *Phase 5 (Naming and defining of themes)* themes were given preliminary names, which should be evocative as well as descriptive, and a short presentation. In *Phase 6 (Writing the report)* the results section was drafted. Although Braun and Clarke recommend an “analytical” style of reporting whereby the results and discussion sections are intermingled [33], this study stuck to reporting consensus and left the part of the analytical work which looked beyond the dataset for a stand-alone discussion section.

## Results

Six focus group interviews were performed, with four to six participants per group. The total number of participants was 33. For participant characteristics, see Table 1. Interviews lasted from 52 to 81 min (median: 69 min).

**Table 1:** Participant characteristics (*n* = 33)

Age	Mean: 50 years (Range: 35–63 years)
Work experience in specialised PC	Mean: 7 years (Range: 0,5–30 years)
Sex	Females: 24 / Men: 9
Professions	Nurses: 15 / Physicians: 13 / Social workers: 3 / Physiotherapists: 1 / Occupational therapists: 1

The analysis resulted in three themes: *Patients have a right to reject information*, *Questioning whether this patient WANTS to reject information* and *There are other values at stake, too*. All themes contained subthemes so that there were a total of seven subthemes – see Table 2 as well as the rest of the results section.

**Table 2:** Themes and subthemes

Patients have a right to reject information		Questioning whether this patient WANTS to reject information		There are other values at stake, too		
*A simple and self-evident right*	*The right must be contextualized*	*Staff must probe patients’ preferences*	*Preferences may change*	*Striving for the good death*	*Staff’s own interests*	*Others’ rights are also relevant*

### Patients have a right to reject information

The core meaning of this theme was that participants looked beyond the case at hand to an abstract, general right *applicable to any patient* to reject information. The first subtheme “A simple and self-evident right” focused on this right itself, whereas the second subtheme “The right must be contextualized” described contextual aspects which might or might not impact on this right. Hence both subthemes indicated that patients such as Mr B have or may have a right such that when he says he does not want to hear more, the PC team should abide by this.

#### A simple and self-evident right

In each focus group interview in this study, there was early and emphatic mention that Mr B has the right to say no to information, or to decide what information to receive. Consider this quote:

*Well I guess that I try to be clear in our conversations, but still never… force anybody to take information they don’t want. To show, like, you have the right to know all that I know, but you also have the right to avoid it.* (Interview V)

The right to reject unwanted information was often mentioned alongside other alleged negative rights, such as rejecting medical treatment, visits by the PC team etc. The right was generally stated as being self-evident, strong and irrefutable. True to its supposedly “self-evident” nature, the right was never further explained. In the few cases where external support was given for the right, this was provided by brief mention of healthcare law and/or coherence with bioethical norms. Mentions of a right to reject unwanted information always met general approval and was never interrogated or directly contradicted by other participants.

Sometimes, Mr B’s alleged right was mentioned in a way which highlighted the team’s responsibilities to honour the right. For instance, a common expression was that staff must “meet the patient where he is at”. This means that his desire not to talk about his health situation is not seen as merely neutral information, but that it implies tangible restrictions on the team’s further communication:

*Perhaps what you need to do is to stop and back up, and then come back. It’s hard to see that you should… what do you call it… go over his defences, force your way, push it. Surely, that must be avoided.* (Interview II)

#### The right must be contextualized

In addition to the self-evident and general right to reject unwanted information discussed above, some participants suggested a right to reject unwanted information which was both contingent and restricted. Many stated that they needed more information about Mr B and his decision to know whether such a right applied. For instance, there were questions about Mr B’s decision making competence (despite the case description stating that Mr B was unequivocally decision competent). Also, there were lively discussions about what might have made Mr B change his mind. It was clear that Mr B’s reasons for changing his mind affected the participants’ views of the ethical challenge here, although participants rarely articulated which reasons would be seen as more conducive to a right to remain uninformed or not. The one exception was that if he had been pressurized by somebody else to remain uniformed, rather than changed his own mind, participants suggested this detracted substantively from the alleged right to remain uninformed. Participants’ provided many and wide ranging speculations about why a patient may be pressurized to change his mind. One participant saw the matter through a cultural lens which affected this participant’s dealing with the matter:

*[If they used joint decisionmaking] this would be easier to handle in a foreign family because we know that, and we have had that before, all of us… but since that’s not really the way Swedes usually behave it would challenge me more [if the family was from a traditional Swedish background]. I’d be more baffled about it (…) I mean it is easier to understand what we don’t understand, right? When we don’t understand a culture it is easier to understand what we don’t understand and accept it.* (Interview III)

Some discussions also situated the specific question of a right to reject unwanted information within the larger framework of patients’ right to be involved in decision-making. According to this view, heeding any particular patient preference was seen as less important than working in a general spirit of shared decision making and balancing decision-making mandates between patient and the PC team. On this view, the relevant right was to be included in the decision-making process along with members of the PC team:

*That they get to say no to something, that’s what’s important. Of course I have a decision at the back of my mind that I’ll make sure to get across later (…) sometimes it’s getting to decide about some little things which makes it feel good [for the patient]* (Interview II).

### Questioning whether this patient WANTS to reject information

This theme explored the way that some arguments did not contradict or balance out a possible right to reject information, but rather questioned whether Mr B did indeed want to reject information. The first subtheme “Staff must probe patients’ preferences” dealt with staff’s difficulty of knowing this, whereas the second subtheme “Preferences may change” instead dealt with, as it were, Mr B’s own difficulty of knowing whether he wanted or did not want information.

#### Staff must probe patients’ preferences

Rather than informing or not informing Mr B, participants stressed that in a case like this the team ought to “probe”, “investigate”, “explore” or “dig deeper”. One thing that they hoped to find by probing was Mr B’s real wishes, which they stated might not at all correspond to the lexical message he had conveyed. The participants asserted that it is not at all uncommon for patients to say one thing and mean another. Suggested reasons for this included that perhaps Mr B did not ever want information, even when he said he did, but rather only said so to please the team or appear brave, or that he still wanted information but now sought to protect somebody else from possibly disheartening information.

Another rationale for probing was the belief that most patients really want to know at least some things about their situation, implying that if a patient claims he wants to know nothing, then probing is required to elucidate what, in fact, he wants to know. The preference for probing over just taking things at face value is expressed as a general rule for communicating in this quote:

*I think it’s pretty dangerous to think you understand everything… you have to kind of ask the question “Is this what you mean?”* (Interview IV).

However, there were also cautionary comments about probing, including that this must never become heavy-handed and that too much curiosity should be kept at bay.

#### Preferences may change

While the previous subtheme dealt with reasons having to do with staff’s difficulty of knowing a patient’s (true) preferences, another factor which made participants cautious to let Mr Br remain uniformed was that Mr B himself might not know what he wanted. Many participants claimed having met patients whose information preferences changed drastically over time, which led participants to stress that information strategies must also be flexible in order to honour changing preferences. Having continuous personal contact with the patient was highlighted as an important advantage in this context. One benefit of continuous contact, to the participants, was that it allowed them to bring the matter up again at a later stage. This was predominantly seen not as a violation of Mr B’s expressed wish, but rather as a communicative tool to make sure that the patient got the information he currently desired:

*Like we already said, it’s a process, so you have to continue later and take it again and adapt to the level where the patient is currently at* (Interview I).

In contrast to the quote above, some participants felt that the team’s previous interaction with Mr B amounted to something akin to a promise to provide him with information. They thus suggested that the previous promise and the present protestation were separate matters which should be balanced against each other.

### There are other values at stake, too

The last theme captured the rich array of reasons and strategies presented by participants, which would go against any possible right to remain uninformed. These reasons for providing information even in the face of Mr B’s protestations were grounded in different things: an ideal (“Striving for the good death”), staff’s interests (“Staff’s own interests”) and third party considerations (“Others’ rights are also relevant”).

#### Striving for the good death

One consideration that seemed to weigh on the participants was that by rejecting information, Mr B might jeopardize his chances of having a good death. There seemed to be consensus among participants across the teams that it was desirable, from the point of view of dying well, that the patient should have understood at least the superficial facts about his situation:

*[Being informed] is a kind of security which increases the possibility of a dignified, calm and good death (…) that you’re not deprived of the time you could have had to think about whether you want to sell your paintings or not, because you didn’t understand how close it was…* (Interview II).

Having a clear picture of the good death, and striving for it, meant the PC teams do not come to the patient’s bedside as blank slates. Instead they have an agenda and goals of care they strive for. The stress on providing sufficient information to the patient was sometimes tied to the patient’s possibility of properly understanding his situation, as in the quote above. Indeed, having the chance to really prepare for death, in terms of saying goodbyes and making final wishes etc., was sometimes expressed as being a bona fide right of its own, which was explicitly pitted against the alleged right of remaining uninformed. On the other hand, providing sufficient information was also tied to the necessity of making medical plans. In this quote, where a participant tries to distinguish the information that should be given from that which could be left out, the desire to make a good plan looms large:

*So I think these are, somehow, different aspects. I’m thinking about how we deal with prognosis, being a palliative team. We may not have an interest, for our sake, to speak about his prognosis and how long time he has left, but we do have an interest in preparing for different things that may happen. So I make a difference there. There are some things that we perhaps don’t push so hard, but other things we do [push]* (Interview VI).

#### Staff’s own interests

It was frequently acknowledged that individual team members and/or the team itself have more personal interests, which may clash with a patient such as Mr B’s expressed wishes. One prominent interest was that the team desired to have done a good job by having helped the patient. This desire was expressed not only in altruistic terms but also in terms of private experience and satisfaction. Helping the patient was largely described in the team’s own terms, in the sense that participants wanted to have provided the help they felt was necessary. Relatedly, the participants expressed a wish for a substantial professional mandate and the manoeuvring space to act as they saw fit. In all interviews, the word “frustrating” or synonyms came up to describe situations such as this, as the patient’s attempt to block information limited staff’s possibility to do their job properly:

*There is a frustration to feel that we cannot do our very best, or we, we could do our best but we aren’t allowed to do our best, what we think is the best. So it’s like a… not that it becomes… you’re not less inclined to help because of it, but it makes it harder, I think.* (Interview II)

Participants wished to keep the flow of communication as free from overt blockages as possible. They expressed a desire to be able to share the information they had and perceived as important. Not being able to share information was described as uncomfortable or that one was being “dishonest” for knowing and not telling.

When the team’s own interests were discussed, there was often hesitation as though the subject was controversial. For instance, there were mixed feelings about staff’s curiosity and their desire to really understand what motivated Mr B to change his mind. Some saw this curious interest as a legitimate aspect of being truly engaged in PC work, whereas others saw over-curious “probing” as something to avoid. Participants often found it challenging to disentangle their private feelings from their professional mandate. Consider this quote where a participant speaks of “converting” patients:

*It is very hard to just accept this, I think… I mean you want to convert them… (…) You don’t want to tell them what to do, neither, you don’t want to do that. But I want to convert them so they have the possibility to… but is that just because I think for myself that’s what would have been good? If he doesn’t think that’s good, like… then it is just what I think he should want to know… sometimes it feels a little like that* (Interview II).

#### Others’ rights are also relevant

As mentioned above, participants worried that others might have influenced Mr B’s decision-making, in a way which might detract from his alleged right to remain uninformed. Additionally, participants expressed concerns that Mr B’s decision might, in turn, affect others. If so, they argued, the effect on others could outweigh his right to remain uninformed. To this participant, the presence of children in the dilemma could make a great difference:

*But I do think that what makes the situation easier, well not easier really but… is if they have small children. Because then there is a responsibility that people have as adults, to protect the children. That is, giving the children what they need. So then you don’t always have the right to say ”No, I don’t want information”, because it also affects someone who cannot govern their own fate* (Interview IV).

## Discussion

The main finding in this qualitative study is that members of PC teams in Sweden find it hard to deal with situations where a patient (“Mr B”) who previously said he wanted information suddenly wishes to remain uninformed. Participants describe this situation as emotionally frustrating, as they perceive the provision of information as a crucial part of their work and they fear that the patient’s attitudes may lead to them not being able to carry out their task as well as they would wish. In this sense, the participants conceive of the patient’s present rejection of information as ill advised. They further describe the situation as a challenging balance where the patient’s right to govern the flow of information is pitted against strong counteracting rationales in favour of giving the information.

The participants in this study strongly endorse, and never straight out question, that patients have a right to choose whether to be informed or not. The possibility of staff ”forcing their way” with information is empathetically rejected, and instead “being flexible” and ”meeting the patient where he is at” were cited as exemplary strategies. This result is well aligned with ambitions in current PC [8, 9, 34]. Yet as participants discuss the alleged right to remain uninformed, they struggle to identify necessary and sufficient conditions for this right. This comes as no surprise, as contemporary bioethics struggles with the same questions. For instance, the issue of how and when family may influence patients’ decision-making without threatening autonomy is much debated [2, 3, 22]. As the participants stress a contextual understanding of autonomy which includes careful attention to Mr B’s decision-making competence and to understanding his real wishes rather than merely what he is saying, they are again mirroring live discussions in contemporary bioethics [11, 35, 36].

For all their endorsement of aspects of autonomy, the participants stated that they would not “just accept” a patient’s plea not to be informed nor pressed on the matter. Counteracting rationales in favour of disclosure weigh heavily in this study. For instance, participants express a desire to be honest to the patient and hide no information. This is in line with ethical recommendations in palliative care [37, 38]. Another important rationale is to be able to make good medical plans, as well as help the patient understand and deal with his situation. Many authors have noted that working for patient preparedness or acceptance of death is a central commitment to PC teams [39, 40]. In ethical parlance, this is a commitment to promoting beneficence, which may tip over into paternalism [41, 42]. This is resonant with findings from other qualitative studies with palliative care personnel, where personnel for instance report struggling to find a balance between “leading and following” [43]. It is in this light the cautiously self-critical comment on “converting” patients in the present study should be read. Participants here communicate a clear picture of the good death, but simultaneously realize they run the risk of sometimes using the patient as a canvas to paint this picture on. Striving for the good death may flow from beneficence concerns as well as self-serving interests, as indicated by results in this material. Thus, there seems to be a risk of staff being smitten by the ”substitute success syndrome” [42], whereby staff may steer a patient in desired directions partly to make themselves feel good.

Another topic where participants self-reflected and expressed self-criticism concerned staff’s own needs and desires. For instance, feeling and expressing curiosity about why Mr B changed his mind was controversial in this study. Many professed their strong desire to explore Mr B’s motivations for changing his mind, and apart from autonomy-honouring reasons to do so there were also self-regarding reasons such as a personal desire to understand other persons. Participants repeatedly pointed out that this may be problematic. Indeed, the recurring concept of “probing” was also invoked as a cautionary note in discussions about understanding Mr B, in the sense of overstepping the boundary of professionalism. All in all, then, it seems to be a slight discord between participants’ ethical reasoning and how they report they would act in a case like Mr B’s. As for professional curiosity, this has been recommended in previous literature as an ideal for PC [44, 45], and a conceptual link between curiosity and empathy has been described [46]. At the same time, the thought that the professional must keep his/her curiosity at bay is commonplace in manuals of professional caring [e.g. 47]. As expressed in this study, the red line between the right and wrong kinds of curiosity in PC may be both important and hard to pick out. This topic clearly merits further investigations.

Participants claimed that the scenario depicted in the case description demanded that staff be “flexible”. Indeed “flexibility” has been described as an ideal in PC [48, 49], and is frequently discussed in relation to practical issues such as scheduling visits [see for instance 50]. Interestingly, participants in this study instead used the notion of “flexibility” to discuss the approach to Mr B’s wish to remain uninformed. As noted above, being “flexible” in the sense of abandoning one’s own agenda and uncritically taking the patient’s perspective was not advocated in this material. Instead, the “flexible” attitudes evidenced here were, first, one where staff reported to “accept” Mr B’s preference while holding on to their conviction that he ought to have agreed to being informed, and second one where staff aim to provide information, but in an underhand or cautious way. Again, the notion of “probing” captures a salient aspect of this study. In regards to the probing approach, participants report consciously using the continuing relations within PC to achieve their goal. The possibility to come back and make a new attempt at providing the information later on eases participants’ stress about the patient’s refusal. In this sense, PC work is conceptualized longitudinally with the passing of time as one tool among many, and relations are seen as unfolding not only in discrete interactions but also over series of interactions. It is in this light that participants’ stress on the importance of building and maintaining an open relationship with patients should be seen [compare 51]. That the slow probing approach would be in violation of Mr B’s desire not to have the issue brought up again was no big source of worry. Instead the sense was that both the medical situation and Mr B’s preferences might very well change over time. A possible topic for future research would be how PC staff understand the notion of “flexibility” and the ways that this ideal informs professional approaches beyond merely practical issues.

Beyond what was said, it is interesting to note also what was *not* said. Although the case involves a clear ethical dilemma, the word “ethics” was rarely mentioned and no participant or group attempted to make a structured analysis of the ethical dilemma. Nonetheless, the participants’ discussions about the right to refuse information and the balancing of this right against counteracting factors is bona fide ethics [52]. Similarly, and perhaps surprisingly, the word “autonomy” was scarce. This could be due to a lack of training in formal ethics, but another interpretation is that the participants did not conceptualize the challenge here as weighing autonomy against beneficence (or similar), but rather as different ways of understanding what autonomy entails. As discussed in the introduction, autonomy is a complex concept and may mean different things in different contexts and according to different people. Indeed, participants explored several autonomy-related questions which are also discussed in theoretical bioethics, such as what makes a decision autonomous [21]. A final possibility is that within PC, the provision of information is commonly seen as a core feature of respecting a patients’ autonomy [53], which may make the thought that *not* providing information could be mandated by autonomy considerations somewhat of a nonstarter.

Although the teams describe the situation as very difficult, the dominant sense in the material is rather that participants are unhappy about Mr B’s preferences than clueless about how to deal with them. For instance, there is no mention of the situation giving rise to moral distress. One reason may be that participants count on time as working in their favour. The participants were self-confident that Mr B would eventually accept information. Hence, awareness of time here seemed to attenuate potential moral distress. This interpretation was supported by the fact that teams admitted there would be greater ethical tension if Mr B had only little time left. Whatever the reason, it would be wrong to construe the ethical qualms witnessed in this material as mere charade. Instead, the recommendations of treading softly and checking and re-checking Mr B’s preferences are motivated by true concern, and they likely represent the participants’ desire to strike a balance between counteracting goals here.

## Methodological considerations

An advantage of the study set-up is that participants were recruited from several regions and belonged to various professions, which likely increased variation in answering patterns. Further advantages included using a well described methodological approach with clear and clearly articulated theoretical assumptions. Furthermore, the results were member checked with members from two groups, which makes for increased credibility.

In this as other focus group interview hierarchies and existing power differentials within groups may hamper participants’ communication [26]. For this particular study, interviewing physicians together with nurses and social workers was nonetheless chosen as this reflected the working circumstances of the PC teams, whose reality the study sought to capture. It should also be pointed out that the study focuses on staff’s *ethical reasoning*, which may not reflect how they in fact act [54]. Another aspect of studying ethical reasoning is that participants’ language may sometimes obstruct as much as it elucidates. For instance, participants in this study placed great weight on a perceived difference between “digging” into Mr B’s reasons for changing his mind, which was scorned, and “probing” the same, which was considered praiseworthy. Although this distinction makes intuitive sense, it is an open question whether it corresponds to any difference to a patient such as Mr B.

The author (JB) being himself an ethicist as well as PC physician, having presuppositions about the topic was unavoidable. More precisely, the author entered the project with the following assumptions: that the vignette case raises ethically difficult questions about autonomy, which different ethical theories would read and resolve differently; that such situations are difficult to manage in the clinic, as the patient’s request creates a self-awareness about the exchange of information; and that staff react to a wish such as Mr B’s in a number of different ways. In addition, self-reflexion before and during this project revealed that the author is ideologically convinced about the value of autonomy as an inspirational ideal in PC, yet still struggle to disentangle the different implications of the ideal. There was (only) one aspect of the analytical work that really triggered the author’s own feelings and that was when staff appeared to simplify the complexity in the situation. Whether this coloured the analysis is difficult to judge.

A potential set-back for this study is that the single author is both interviewer and analyst. Although such studies are acceptable within the tradition of reflexive thematic analysis [27], there is a risk that the single analyst becomes blind to potentially relevant features of the data. During the analysis phase the author used another researcher (Niklas Juth) as a “critical friend”, asking for Juth’s view on some interpretative matters regarding the transcripts and how to analyse the relationships among themes. His input was no substitute for real co-authorship but nevertheless valueable in the process.

## Conclusion

In this qualitative study of how PC staff reason about a fictional case in which a patient first expresses a wish to be informed, but then says he does not want information, the main finding is that staff find it hard to “just accept” his preference to remain uninformed. Participants express support for an abstract or contextualized right to remain uninformed, yet pit this against countervailing considerations that, ultimately, are seen as overriding. Through the aid of time and flexibility, participants state that they will likely be able to communicate relevant information without “forcing their way”. The results are instructive in understanding the views of PC staff in regards to autonomy, changing patient preferences, the value of information and professional attitudes. As the themes were engaging as well as challenging to participants in this study, PC teams may do well to discuss the implications of patient autonomy more. Further studies on the topic could explore the meaning of “flexibility” within palliative care, as well as the observed controversies surrounding staff’s private interests, including curiosity and the wish to do a good job.

### Electronic supplementary material

Below is the link to the electronic supplementary material.

Supplementary Material 1

## Data Availability

The data generated and analyzed in this study is not publicly available due to ethics restrictions, but is available from the corresponding author on reasonable request.

## Footnotes

1 Forthcoming from the same author is ”Autonomy of the dying – charting the palliative care ethos”.

## Abbreviations

PC: Palliative care
