# Supplementary material for: “It is very hard to just accept this” – a qualitative study of palliative care teams’ ethical reasoning when patients do not want information
Source: BMC Palliat Care. 2024 Apr 5;23:91. doi: 10.1186/s12904-024-01412-8 (PMC10996159; doi:10.1186/s12904-024-01412-8)
Supplement: Supplementary file 1 — Supplementary Material 1 [file 12904_2024_1412_MOESM1_ESM.docx]

(Interview commences with interviewer reading the following vignette):

*Mr B, a man with disseminated prostate cancer, is cared for by the specialized palliative care team. He has repeatedly stated to the team that it is vital to him to have full knowledge about his situation, including what his death may come to look like. As there have been many bodily symptoms to deal with, his questions about death and dying have not yet been answered. Now, the team has set aside time for the conversation Mr B has asked for. But as soon as they start talking, Mr B states that he does not want any more information and that this issue should not be brought up again. He provides no reason. Nothing in the situation appears to have changed. He is perceived to be of sound mind, now as before.*

- What do you think when you hear this?
- Do you recognize this kind of situation?
- What is ethically important in a situation such as this?
- What kind of considerations would you make in a situation such as this?
- How would you normally deal with this kind of situation?
- What might you, as staff, feel in a situation such as this?
- Do you have other comments?
